# Structural Basis of Directional Switching by the Bacterial Flagellum

**DOI:** 10.21203/rs.3.rs-3417165/v1

**Published:** 2023-10-23

**Authors:** Steven Johnson, Justin C. Deme, Emily J. Furlong, Joseph J.E. Caesar, Fabienne F.V. Chevance, Kelly T. Hughes, Susan M. Lea

**Affiliations:** 1 Center for Structural Biology, CCR, NCI, Frederick, MD 21702-1201 USA; 2 Sir William Dunn School of Pathology, University of Oxford, Oxford, UK.; 3 Central Oxford Structural Molecular Imaging Centre, University of Oxford, Oxford, UK.; 4 Department of Biology, University of Utah, Salt Lake City, Utah, USA.

## Abstract

The bacterial flagellum is a macromolecular protein complex that harvests energy from ion-flow across the inner membrane to power bacterial swimming in viscous fluids via rotation of the flagellar filament. Bacteria such as *Salmonella enterica* are capable of bi-directional flagellar rotation even though ion flow is uni-directional. How uni-directional ion-movement through the inner membrane is utilized by this macromolecular machine to drive bi-directional flagellar rotation is not understood, but a chemotactic response regulator in the cytoplasm is known to reverse the direction of rotation. We here present cryo-EM structures of intact Salmonella flagellar basal bodies, including the cytoplasmic complexes required for power transmission, in conformations representing both directions of rotation. The structures reveal that the conformational changes required for switching the direction of rotation involve 180 degree rotations of both the N- and C-terminal domains of the FliG protein. Combining these models with a new, high-resolution, cryo-EM structure of the MotA_5_B_2_ stator, in complex with the C-terminal domain of FliG, reveals how uni-directional ion-flow across the inner membrane is used to accomplish bi-directional rotation of the flagellum.

The bacterial flagellum is the key organelle that responds to signals from the chemotaxis machinery to permit directional motility of the cell^1–3^. Flagella exist in a wide range of bacterial species, including Gram-negative and Gram-positive species, and display a large degree of compositional and structural variation in the outer regions, with adaptations permitting periplasmic flagella and both membrane sheathed and unsheathed extracellular filaments^4^. The huge variability is driven by the differing environments experienced by the bacteria and the need for different levels of torque. However, the core motor components that are tethered to the inner membrane are highly conserved across all species, with the main variation being in stoichiometry rather than composition. The central rotating unit, the rotor, consists of the inner membrane (IM) resident MS-ring and the cytoplasmic C-ring^5^ (Fig. 1a). The MS-ring functions as a structural adaptor^6^, interacting with the C-ring that coordinates with peripheral stators to generate torque^7^, and with the export gate responsible for secretion of the axial components of the rod and flagellar filaments^8^.

Flagellated bacteria use ion-motive force driven rotation of the flagellar filament to swim^9^ in a series of short, straight runs, with directional reorientation occurring at the end of each run^3^. The exact details of the reorientation event vary between species, but always involve binding of the phosphorylated form of a small chemotaxis regulator, CheY, to the cytoplasmic portion of the flagellar basal body^10^. In peritrichous bacteria, including *Salmonella enterica*, CheY-P binding results in full reversal of the direction of rotation of the flagellum, from the default counter-clockwise (CCW) rotation to clockwise (CW) rotation. This in turns shifts the cell from swimming (CCW) to tumbling and reorienting (CW) (Fig. 1a).

The flagellum is therefore a reversible biological rotary motor organized into two essential components: a central rotor and a peripheral stator system. This nomenclature is historic, based on analogy to electrically driven motors, whereby proton flow through the “fixed” stator components propagate rotation in the rotor. However, recent structural work on the stators has revealed that they are themselves likely rotary motors, and that proton flow drives rotation of a MotA pentamer around a fixed MotB dimer^11,12^. Therefore, rotation of the central flagellar basal body is akin to a large cog being driven by a series of smaller motor cogs. In addition to rotary dynamics, stators are also laterally dynamic^13^, with recruitment being linked to the load on the flagellum^14,15^. At low load a single stator “cog” is sufficient to drive the larger C-ring “cog”, and increased load leads to up to 11 stators driving a *Salmonella* flagellum^16^. Stators are also mechano-sensitive, opening their channels in response to force applied upon binding of domains of MotB to structures in the periplasm^17^.

The C-ring, or “switch complex”, consists of around 34 copies of a FliG/FliM/FliN sub-complex in *Salmonella*^18,19^, which nucleates onto the 34 copies of the MS-ring cytoplasmic tail. Low resolution cryo-electron microscopy (cryo-EM)^20^ and cryo-electron tomography (cryo-ET)^21,22^ studies, combined with crystal structures of isolated proteins and domain (reviewed in ^23^,^24^), have suggested possible arrangements of the C-ring but have lacked the detail to allow confident docking of subunits, and hence provided limited insight into the molecular details of rotational switching events. The only known regulator of rotation direction, CheY-P binds to the FliM component of C-ring^25^ and therefore rotation reversal is triggered by alterations to the larger, “passive”, cog, not via changes to the direction of the “active” stator cog. Binding of CheY-P to FliM has been proposed to lead to changes in the orientation of the FliG domain thought to bind to the stator^23,24^, but such details have not previously been observed in a functional rotor at an interpretable resolution. Rotor reversal is also highly sensitive, with a Hill coefficient of up to 21 with respect to CheY-P concentration^26^, consistent with observations that full occupancy of CheY-P on FliM is not required for switching^27^. However, how such cooperatively could be achieved is unclear.

To gain mechanistic insight into the process of directional switching, we determined cryo-EM structures of of *S.* Typhimurium basal bodies with intact C-rings trapped in the CCW and CW states (Figs 1,2). The maps ranged in resolution from 3.2 – 5.5 Å, allowing *de novo* building of the core domains and placement of Alphafold2 models of more mobile regions. The structures reveal an extraordinary degree of intra-subunit and inter-subunit intercalation that likely contribute to the high degree of cooperativity of the switching event. We have also determined structures of a complex between a stator and the FliG domain (FliG_C_) that provides the point of contact between the active and passive cogs. This allows us to understand how the arrangement of the cogs flips from having the active cog on the outside to the inside of the passive C-ring cog, so explaining how a uni-directionally rotating active cog can drive the passive C-ring cog bidirectionally.

## Conserved Architecture of CCW and CW locked C-rings

**Extended Data Figure 1.**
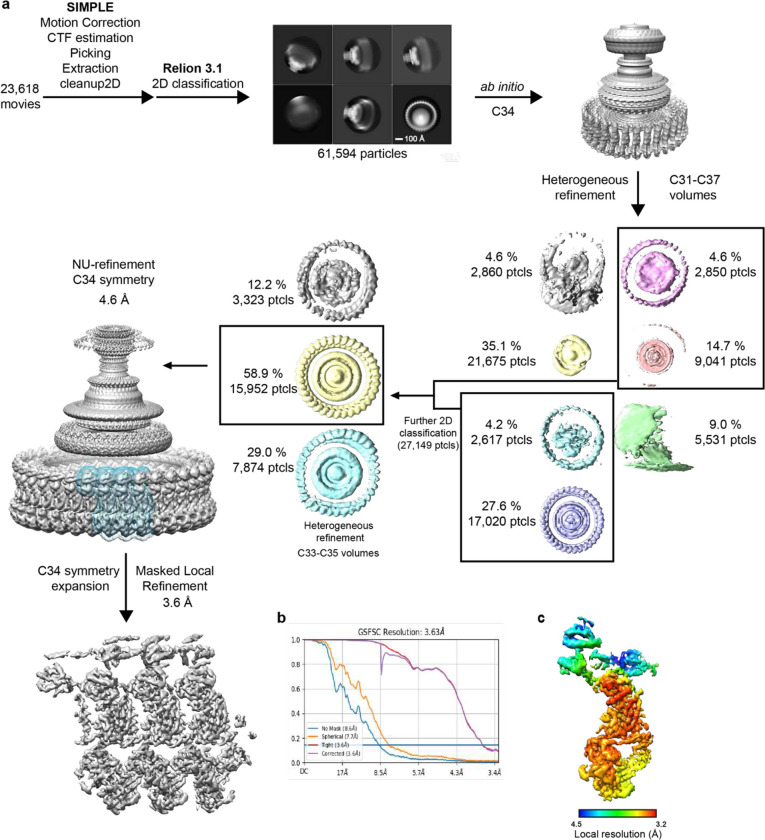
Cryo-EM processing workflow, showing local and global map quality for the CCW C-ring structure. **a**, Image processing workflow for the CCW C-ring. **b**, Gold-standard FSC curves used for global-resolution estimates within cryoSPARC. **c**, Local-resolution estimation of reconstructed map as determined within cryoSPARC.

**Extended Data Figure 2.**
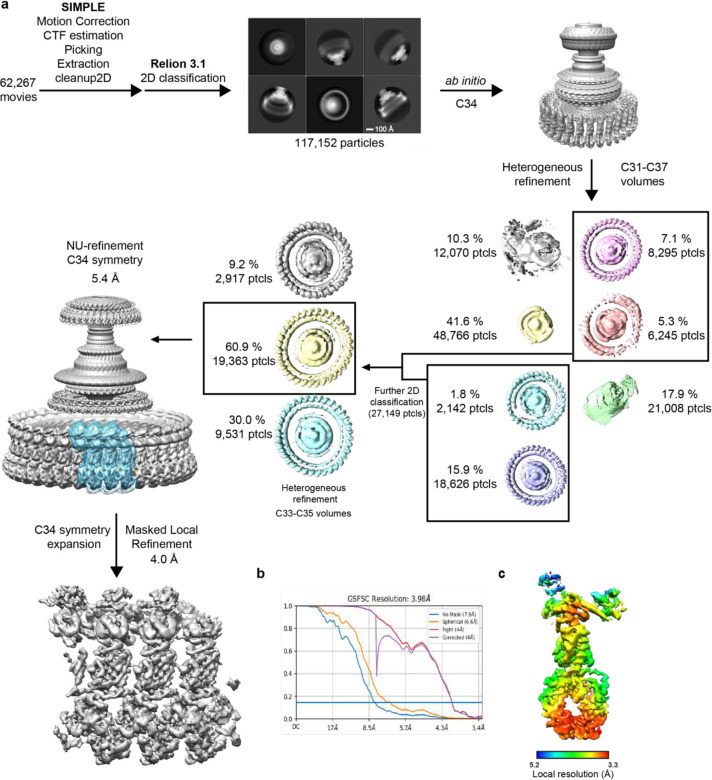
Cryo-EM processing workflow, showing local and global map quality for the CW C-ring structure. **a**, Image processing workflow for the CW C-ring. **b**, Gold-standard FSC curves used for global-resolution estimates within cryoSPARC. **c**, Local-resolution estimation of reconstructed map as determined within cryoSPARC.

**Extended Data Figure 3.**
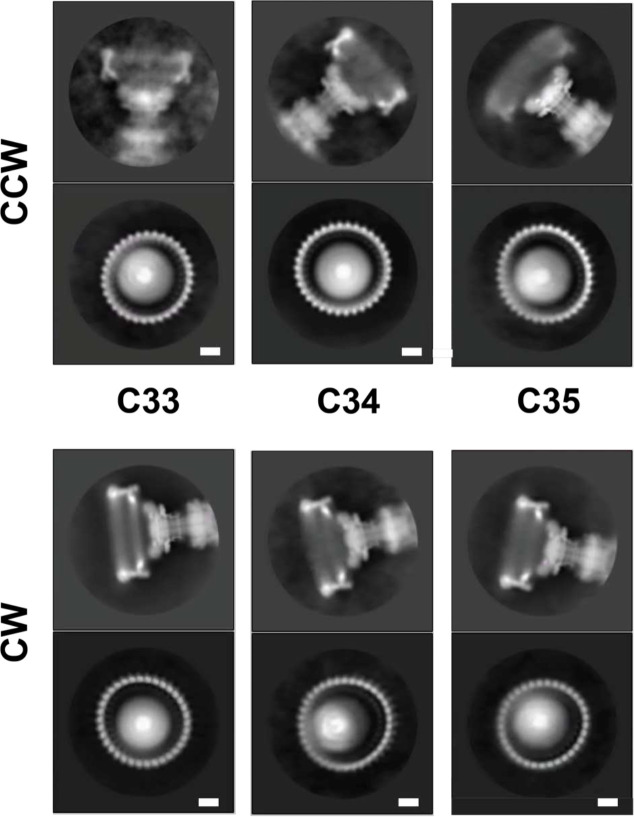
Different Symmetries are apparent in 2D classes following particle classification in 3D 2D class averages are shown for CCW and CW particles separated into different symmetries via 3D classification. An example of a side-view is shown above and a top-down view below. The subunit numbers in the top-down views can be counted to reveal a symmetry consistent with the 3D classification.

**Extended Data Figure 4.**
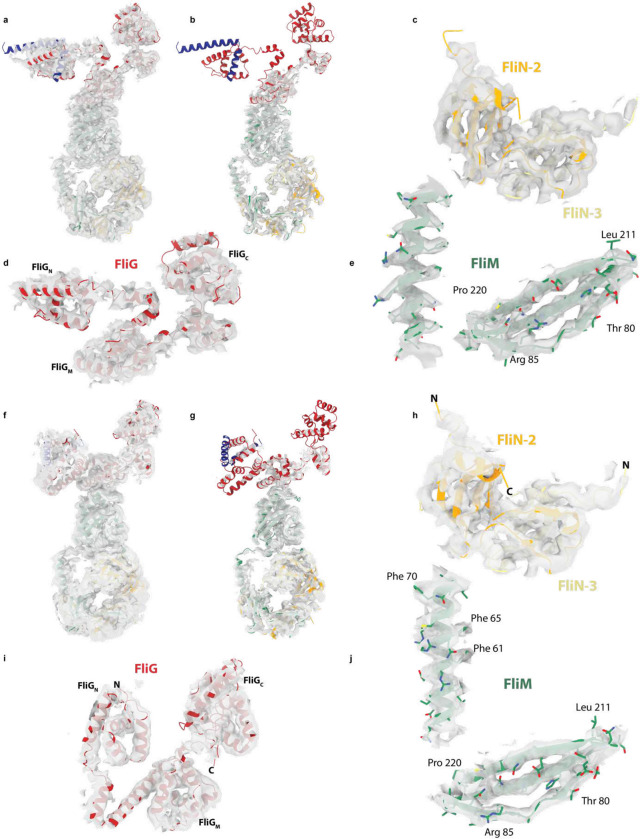
Fit of coordinates to CCW and CW cryoEM volumes **a-e**, show different views at different contour levesl of the CCW coordinates within the CCW volume. f-j, show the same for the CCW volume. **a,b** and **f,g** show the full subunits (CCW and CW respectively) at a lower (**a,f**) and higer (**b,g**) contour levels revealing the FliG domains at the top of the subunit are the most mobile regions. **c,h** depict the volume surrounding two of the three FliN domains, **d,i** the volume around FliG and **e,j** for two regions of FliM, all for CCW and CW respectively.

**Figure 1.**
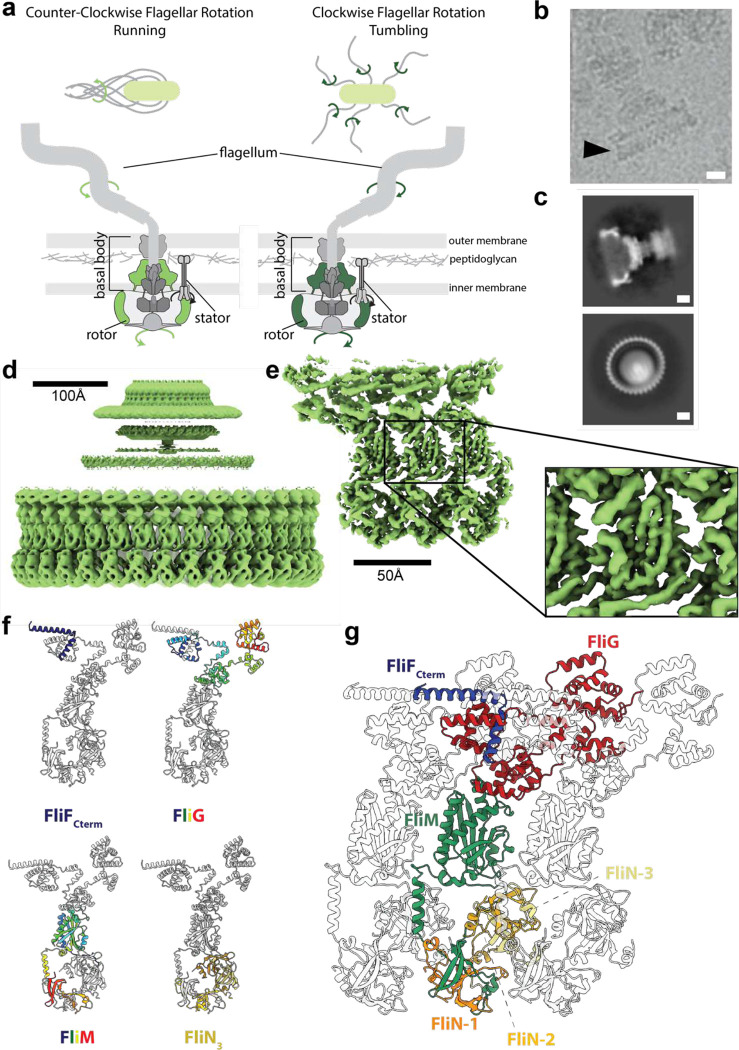
Structure of the Counter-Clockwise rotating state of the *Salmonella* Typhimurium flagellar C-ring. **a,** Upper panels: counter-clockwise (CCW) rotation of the Salmonella flagellum leads to flagellar bundling and coordinated movement whilst clockwise (CW) rotation leads to uncoordinated tumbling. Lower panels: cartoons of the flagellar assembly across the bacterial membranes highlighting in green (CCW light green, CW dark green) the location of the rotating structures (the rotor composed of the MS- and C-rings). **b,** Region of a cryoEM micrograph showing a flagellar basal body with intact C-ring in an approximately side on view (CCW state). Scale bar 100 Å. **c,** 2D class averages (CCW state) show both ‘side’ (upper panel) and top-down (lower panel) views. In the top-down view the 34 C-ring subunits can be counted. Scale bar 100 Å. **d,** 34-fold symmetrized cryoEM volume (light green) of the CCW locked flagellar rotor with alignments focused on the C-ring reveals the domain organization of the C-ring (lower density), and that the MS-ring is not aligned with the C-ring (blurred upper density). **e,** Using a mask around 3 subunits and refining symmetry expanded particles generates a higher resolution volume (3.2–4.5 Å across the subunit) for the central subunit allowing building and refinement of atomic models. **f,** A cartoon representation of a single subunit is shown highlighting the locations of the different protein chains. FliG and FliM are shown using rainbow coloring (blue at N-term to red at C-term), the cytoplasmic C-terminus of FliG in dark blue and the three copies of FliN in different shades of yellow. **g,** Cartoon representation of 3 subunits from a 34-mer C-ring. The central subunit is colored by protein chain with FliF and FliN colored as in (**f**) with FliG-red, FliM-green, the neighboring subunits in grey.

**Figure 2.**
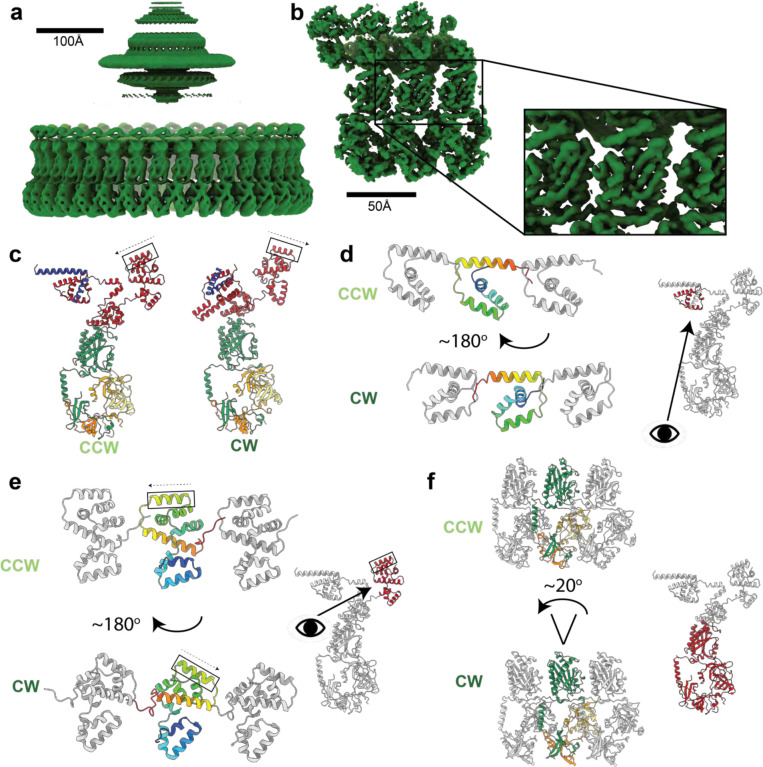
Structure of the Clockwise rotating state of the *Salmonella* typhimurium flagellar C-ring and large domain rearrangements between the CCW and CW states. **a,** 34-fold symmetrized cryoEM volume (dark green) of the CW locked flagellar rotor with alignments focused on the C-ring reveals, as shown for in Figure 1d for the CCW state, the domain organization of the C-ring (lower density), and that the MS-ring is not aligned with the C-ring (blurred upper density). **b,** Using a mask around 3 subunits and refining symmetry expanded particles generates a higher resolution volume (3.3–5.2 Å across the subunit) for the central subunit allowing building and refinement of atomic models. **c,** A cartoon representation of single subunits colored as in Figure 1g shows the dramatic changes in domain arrangements between the CCW and CW states. The FliG_C_ torque helix is boxed and the arrow indicates the N-C direction. **d-f** highlight the dramatic domain rearrangements domain by domain for the subunit regions indicated in red on the cartoon of the single full subunit in each panel. The domains are shown in context of the 3-subunits with the CCW state above and the CW state below (central subunit domain color - rainbow (**d,e**); by chain as in c (**f**)). (**d,e)** The eye and arrow show the approximate view point from which the CCW/CW domains are viewed, (**f**) view is as for the small, full subunit.

*S.* Typhimurium basal bodies with intact C-rings (Fig. 1b) isolated from strains manipulated to lock them in either the CCW (Δ*cheY*) or CW (*fliG-*Δ*PAA*) states revealed heterogeneity in the region of the C-ring, with top-down views being blurred on one side. Initial maps were calculated by applying C34 symmetry on the assumption that the C-ring matched the symmetry of the MS-ring, and these were then used to calculate volumes with C31-C37 symmetry for use in supervised 3D classification (Extended Data Figs. 1,2) . In both CCW and CW samples, variation in subunit stoichiometry was observed consistent with earlier low-resolution observations^20,28^, with C34 being the dominant species. 2D analysis of the classified particle sets revealed top-down C-rings where subunit numbers could be counted (Fig. 1c, Extended Data Fig. 3), validating the classification protocol. Refinements of the whole C-ring with C34 symmetry applied produced interpretable 4.6 Å and 5.4 Å maps in the CCW and CW forms respectively, where secondary structure was especially clear in the FliM_M_ region of the map (Figs 1d,e and 2a,b). To investigate if resolution was limited by local variation in the subunit orientation, i.e. to allow for the C-ring not being a perfectly symmetric object, we performed symmetry expansion and then local refinement around 1–3 subunits. This improved the resolution of the whole subunit, with the FliM_M_ region reaching 3.2 Å (CCW, Fig. 1e) and 3.3 Å (CW, Fig 2b), with clear sidechain detail. Resolution was lowest (~ 5 Å) in the regions of FliG_N_ and FliG_C_, but the clear secondary structure elements observed still allowed unambiguous placement of Alphafold2 models of the domains (Extended Data Fig 4).

**Extended Data Figure 5.**
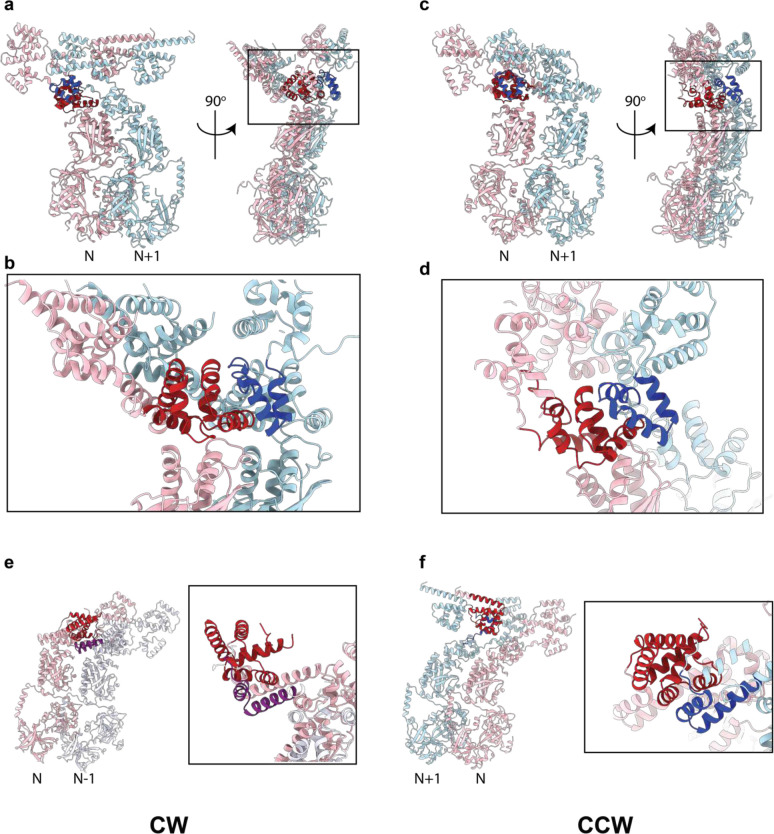
Domain swaps between C-ring subunits involving regions of FliG Two neighbouring C-ring subunits are shown in cartoon representation coloured pink (copy N) and either light blue (N+1) or lavender (N−1). **a,b,e**, are from the CW assembly and **c,d,e** from the CCW. **a-d**, depict the domain swap to assemble the FliG_M_ domain (dark red and dark blue to denote which subunit the sequences originate in). **e-f** depict the domain swap to assemble FliG_N_ (coloured dark red and purple (**e**) or dark red and dark blue (**f**)).

**Extended Data Figure 6.**
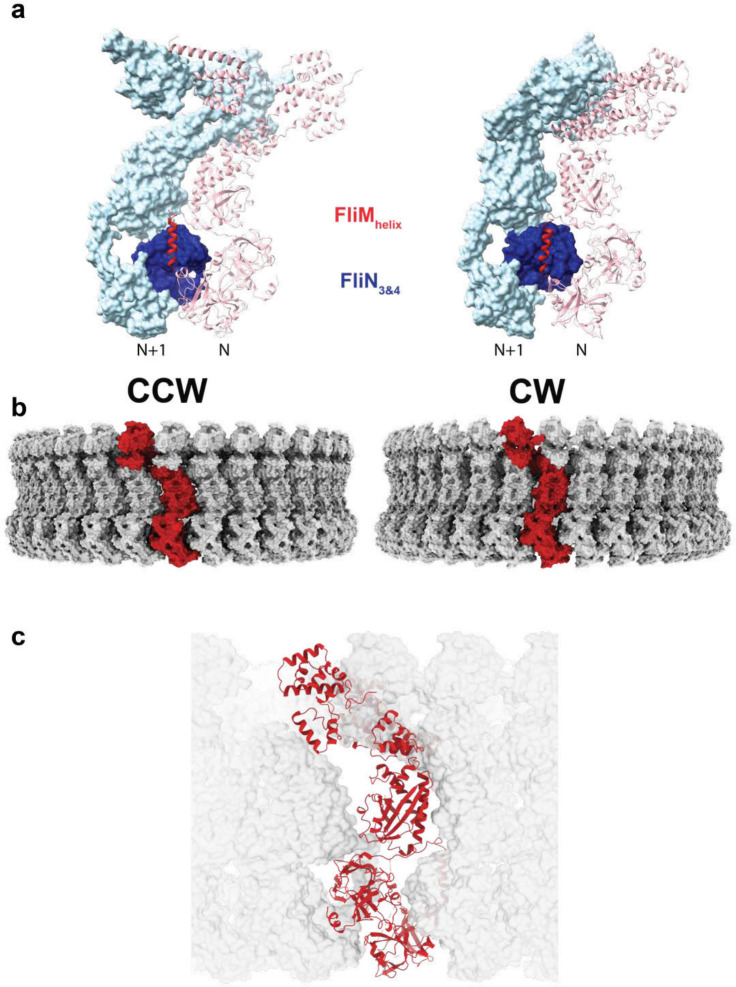
Complexity of Subunit Packing within the CCW and CW C-rings **a,** An unanticipated packing between a secondary structural element immediately following the FliM_M_ domain leads to further inter-subunit packing interactions between FliM and FliN in addition to the previously proposed lock-washer interactions. This new element occurs in both states with subtly different contacts. **b,** A single subunit is colored red in the context of the C34 C-ring in both states to emphasise how the vertical subunits visible in previous low resolution volumes are constructed from domains originated in multiple subunits. **c,** A subunit taken from a CW state (red ribbon) is incompatible with packing between subunits in the CCW states reinforcing the cooperativity in switching states that must exist.

**Figure 3.**
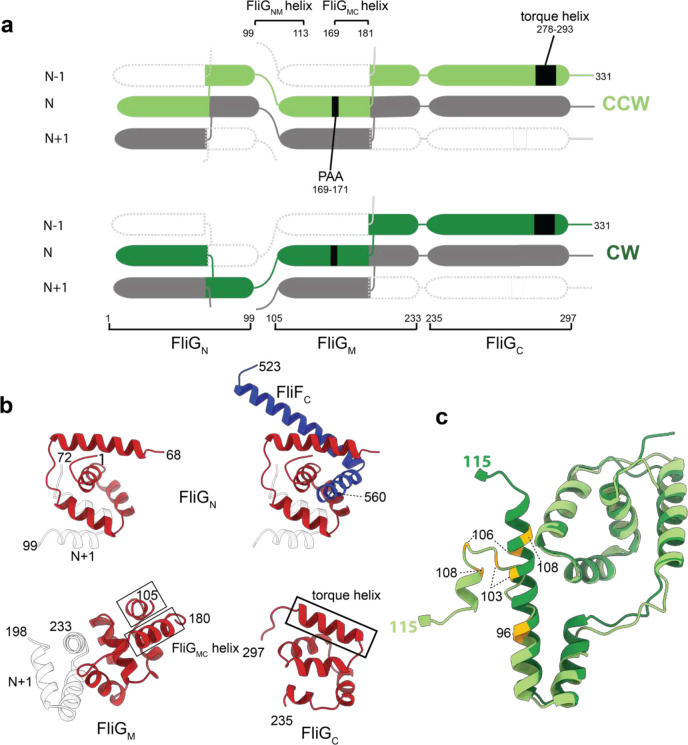
Both FliG_N_ and FliG_M_ domains are completed via inter-subunit domain swaps but FliG_N_ inverts which subunit contributes the C-terminal portion of the fold in the CCW and CW states. **a,** domain swapping within FliG is shown with residue boundaries and key features discussed in text indicated for both CCW (upper) and CW (lower) states. **b,** Each FliG domain is shown with the N-subunit derived sequence shown in red and the N+1 in grey for the CW state. FliG_N_ is shown twice in the upper half of the panel with FliG residues on the left-hand side and including the FliF_C_ that folds with these sequences to complete the domain on the right-hand side. **c,** Resdiues 1–115 of FliG are shown in CCW (light green) and CW (dark green) states. Residues that introduce a strong clockwise bias are colored yellow.

The overall properties of the C-ring structure are conserved between the two rotational states. The individual subunits of the C-ring are built from FliF_C_:FliG:FliM:FliN_3_ complexes (Figs 1f,g, 2c). The membrane proximal region is formed from FliG protomers, tethered via a FliF C-terminus that forms a core part of the G_N_ domain fold (Fig. 1f,g). The FliG_M_ domain forms the main interaction with a layer of FliM protomers underneath the FliG ring, and the most membrane distal layer is formed from a continuous ring of FliM_C_:FliN_3_ sub-complexes (Fig. 1g). C34 is the most populated symmetry of both states (Extended data Figs. 1,2). Lateral contacts in both CCW and CW states are dominated by domain swaps at all major layers, many of which involve domains of FliG (Figure 3, Extended Data Fig. 5). At the top of FliM_M_, the FliG_M_:FliM_M_ interaction interface is consistent with that seen in previous various crystal structures (Figs 1f, 2c), with the FliG_M_ domain being formed via inter-molecular stacking of residues at either end of the FliG_MC_ sequence(Fig. 3a,b). In both CCW and CW states the FliG_M_ domain is formed by residues 105–180 of copy N stacking against residues 198–233 of copy N+1 (Fig. 3a,b, Extended Data Fig. 5a–d), as seen in crystal structures of FliG and its isolated domains (reviewed in ^29^. Interestingly, a near identical domain swap forms the FliG_N_ domain that packs to form the inner most layer (Fig. 3a,b, Extended Data Fig. 5e,f). Residues 523–560 of the C-terminus of FliF fold with residues 1–68 of FliG to form a sub-domain homologous to both FliG_M_ and FliG_C_, and this in turn packs against residues 69–104 of the neighboring copy. At the base of the structure, the C-terminus of FliM forms a heterodimer with one copy of FliN and then packs against a FliN homo-dimer, in the “lock-washer” arrangement proposed in previous studies^20,30^. Previously unobserved structural elements include the FliM helix immediately after FliM_M_ which packs against a neighboring subunit FliN dimer (Extended Data Fig. 6). The N-terminus of one copy of FliN also wraps around the helix to some degree. The other interface of the FliN dimer binds the N-terminus of the FliN that is paired with FliM_C_.

## Structural differences between CCW and CW locked C-rings

**Extended Data Figure 7.**
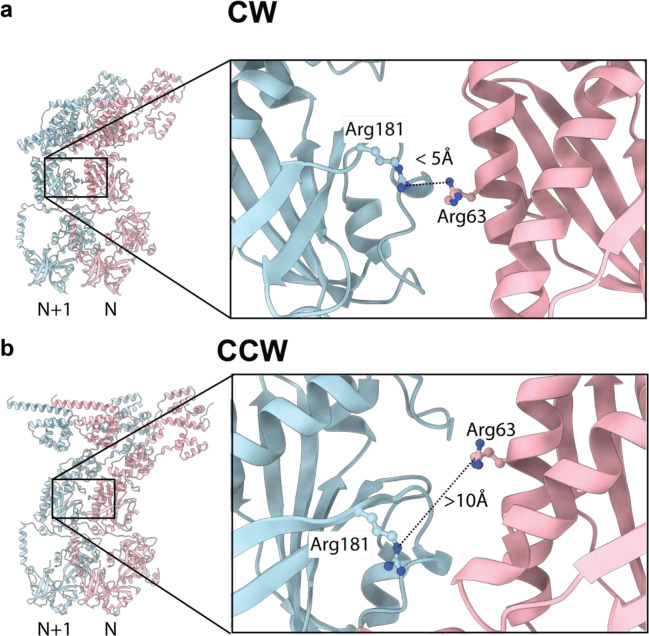
FliM arginines that favour CW rotation when mutated are proximal in the CW but not the CCW state **a,** FliM Arginine 63 and 181 from the N and N+1 subunits respectively, are proximal to each other at the subunit interface in the CW state, **b,** but are separated in the CCW state.

As seen in lower resolution tomographic studies from other organisms^21,22^, there is a change in the outer perimeter wall of FliG_M_:FliM_M_:FliM_C_:FliN_3_, with the CW conformation being more upright than the CCW (Fig. 2c). The FliG_M_:FliM_M_ domains rotate through an angle of 22 degrees as a rigid object (Fig 2f), while the FliM_C_:FliN_3_ spiral pulls up slightly and hinges inwards relative to FliM_M_. The ability to undergo this movement is facilitated by relatively sparse contacts between adjacent FliM_M_ domains (Extended Data Fig. 6), though the domain faces presented in each state are consistent with the extensive cross-linking that has been carried out in these domains^31^. Mutations that impact switching and lead to CCW-locked and CW-locked states also map to these faces of FliM_M_ (reviewed in ^31^. Interestingly, a pair of arginines (R63, R181) that favor CW rotation when mutated face each other in the CW state, but not the CCW state (Extended Data Fig. 7), suggesting that electrostatic repulsion may play a key role in this region of the structure when it comes to switching.

**Extended Data Figure 8.**
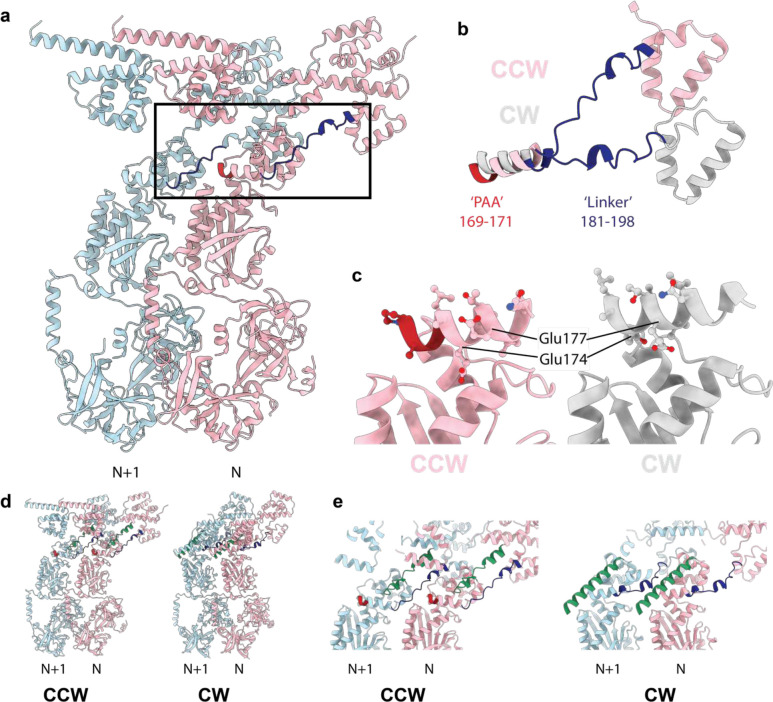
Structural Implications of the PAA CW-locking mutation **a,** Two subunits in the CCW states are shown colored light pink (N) and light blue (N+1) with the FliG PAA sequence that, when deleted, locks the C-ring in the CW state highlighted in dark red and the FliG linker between FliG_M_ and FliG_C_ highlighted in dark blue. **b,** When the FliG_M_ domains are used to overlay the CCW (light pink) and CW (silver) states the deletion of the PAA sequence (dark red in the CW state) leads to a pulling-up of that helix and reorientation of the FliG_M_-FliG_C_ linker. **c,** overlaying the CCW (light pink) and CW (silver) by matching of the FliM_M_ domain reveals how the FliG_M_ helix containing the PAA sequence (dark red in the CCW state), is reoriented altering the side chains presented for interaction with the FliM_M_ domain below. **d-e,** the linker between the FliGM and FliGC domains (green cartoon) is also in the inter-subunit interface and reorients becoming more helical in switching between CCW and CW states. **d,** shows full cartoon view of two neighbouring subunits in CCW (LHS) and CW (RHS) states with the PAA highlighted in red, the FliGM-FliGN linker in dark blue and the FliGM-FliGC linker in green. **e,** shows a closeup slab removing overlaying elements colored in the same way.

A significant difference is observed in the FliG_M_ domain, where the CW-locking PAA deletion at residues 169–171 leads to a shift of the FliG_MC_ helix by almost a full helical turn (Extended Data Fig. 8). The shift does change some of the contacts at the FliG_M_:FliM_M_ interface that likely explain the reported lower affinity of this interaction in the CW-locked mutant^32^, but the helix remains bound to FliM_M_ (Extended Data Fig. 8a–c). The C-terminal end of the FliG_MC_ helix leads into a less well-ordered linker that connects the two domain-swapped portions of the FliG_M_ domain, and this is a major hinge point between the two states (Extended Data Fig. 8d,e). This linker in turn contacts the PAA sequence and the loop preceding it from the neighboring copy (Extended Data Fig. 8d,e), thereby highlighting the importance of this region of the structure in tying the ring together and propagating lateral structural changes. Also at the C-terminus of FliG_MC_ is a major contact to the helix that links FliG_N_ and FliG_M_, termed FliG_NM_. Here there is a major structure change in FliG_NM_ between the two states. In the CCW form, FliG_NM_ folds back through 180 degrees via a turn involving residues 101–106, whereas the CW form is a straight helix that runs from 87 to 112 (Fig. 3c). This leads to the entire FliG_N_ domain rotating through 180 degrees between the two states (Fig. 2d). The FliG_N_ domain maintains its overall domain swapped conformation, but residues 72–99 make contact with N−1 rather than N+1 (Fig. 3a). Many mutations that are seen to induce a strong clockwise bias (E95D, D96V/Y, T103S, G106A/C, E108K) are localized to FliG_NM_^33^, with many clustered around the hinge point and FliG_MC_ interaction point (Fig. 3c), suggesting that this structural change is one of the key driving forces of switching.

**Extended Data Figure 9.**
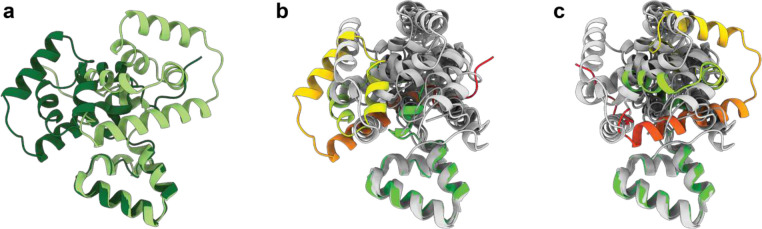
Overlays of the FliG_C_/FliG_M_ packing with prior crystal structures reveals the arrangement seen in both C-ring assemblies is unique. **a**, The arrangement of the FliG_C_ (residues 234–331) differs by a rotation of 180° between the CCW (light green) and CW (dark green) structures relative to FliG_M_ (residues 198–233 shown at bottom of panels and used to generate overlays). **b-c**,Previous crystal structures of FliG_C_/FliG_M_ have revealed a variety of different arrangements between the domains. Earlier crystal structures (PDB ids 3ajc, 1lkv, 3usw and 3usy (two chains independently overlaid)) were overlaid onto the CCW (panel **b**) and CW (panel **c**) FliG_M_-198–233 using matchmaker within ChimeraX. None of the earlier crystal structures place the C subdomain in either position seen within the C-ring states.

**Extended Data Figure 10.**
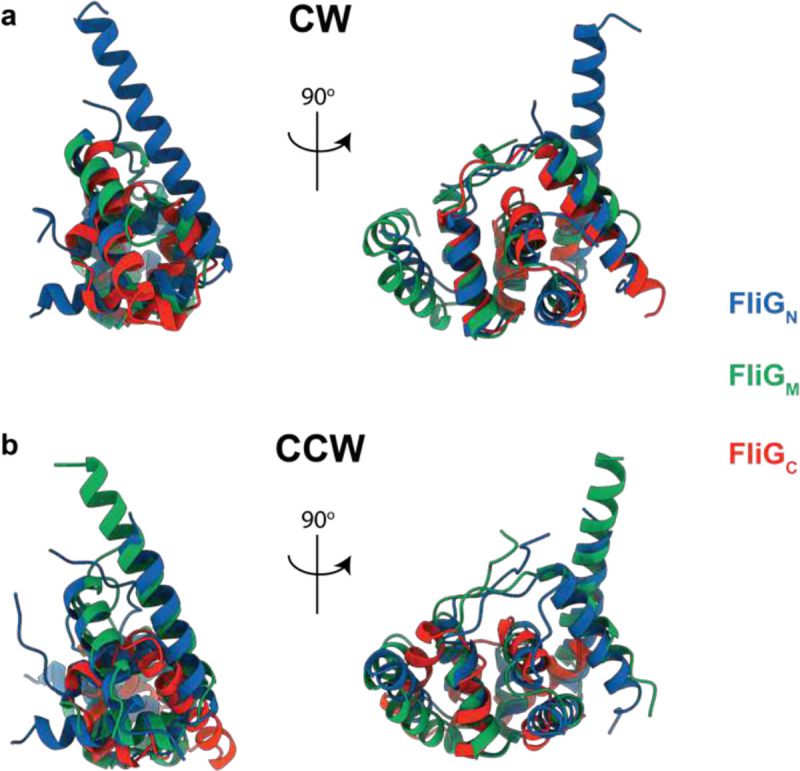
The three FliG Domains assembled via inter-subunit domain swaps share a common architecture The compound FliG_N_, FliG_M_ and FliG_C_ domains assembled via inter-subunit domain swaps share the same domain architecture in both the CCW (**a** – R.M.S.D. 2.3 +/− 0.4 Å) and CW (**b** – R.M.S.D. 2.4 +/− 0.2 Å) states R.M.S.D. each domain onto all others, both states, 2.1 +/− 0.6 Å. Two views of the overlaid domains in a cartoon representation are shown for each state with the domains coloured as shown in the key.

At the top of the structure, the FliG_C_ domain also undergoes a 180 degree between the CCW and CW states (Fig 2e). This rotation occurs at M233 of the conserved MFLF motif. However, neither conformation of FliG_C_ relative to FliG_M_ matches those observed in prior crystal structures (Extended Data Fig. 9). Overlay of all of the available FliG_C_ /FliG_M_ structures demonstrates a large sampling of conformational space at this interface, suggesting that the relative orientation of these domains is likely determined by other factors (Extended Data Fig. 9). In both CCW and CW conformations, the C terminal residues of FliG packs against the neighboring FliG_C_, likely stabilizing the observed structures (Fig. 2e). Irrespective of which rotational state and direction of domain swapping, the FliG_N_, FliG_M_ and FliG_C_ are structurally highly conserved with overlays between all three domains in both CCW and CW states showing only a R.M.S.D. of 2.1 +/− 0.6 Å (Extended Data Fig. 10).

## Molecular detail of stator C-ring interactions

**Extended Data Figure 11.**
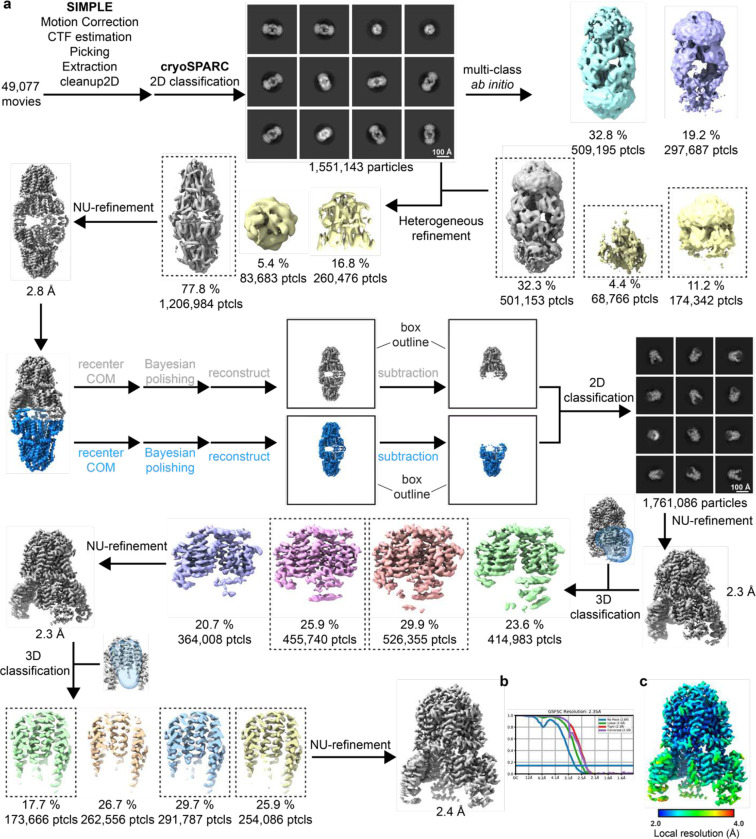
Cryo-EM processing workflow, showing local and global map quality for MotAB + FliG_c_. **a**, Image processing workflow for MotAB + FliG_c_. **b**, Gold-standard FSC curves used for global-resolution estimates within cryoSPARC. **c**, Local-resolution estimation of reconstructed map as determined within cryoSPARC.

**Extended Data Figure 12.**
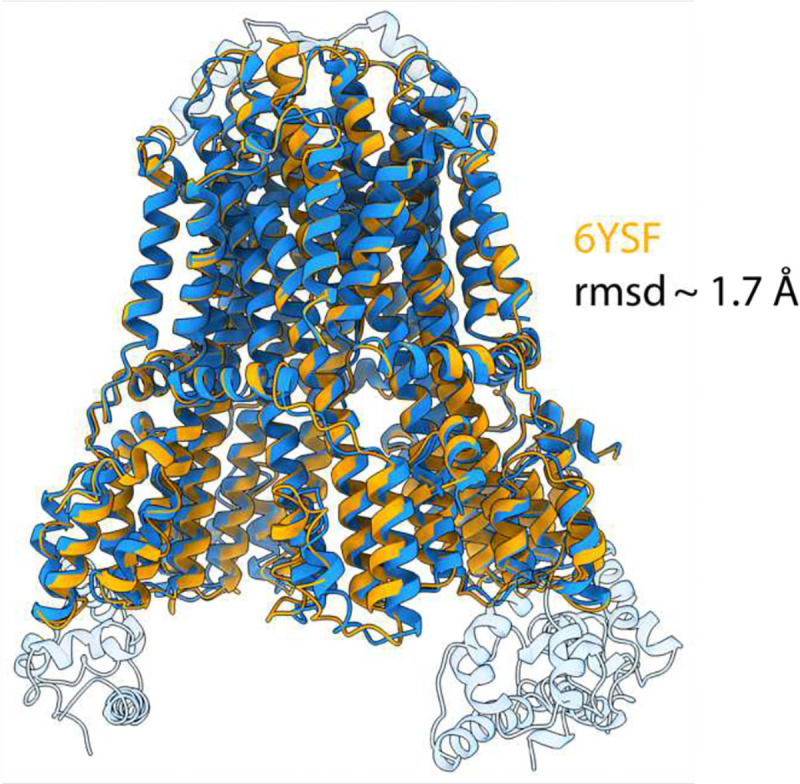
Structural alignment of *C. sporogenes* MotAB with FliG-bound MotAB. *C. sporogenes* MotAB (PDB: 6YSF) superposed with FliG-bound MotAB structure presented in this study. MotAB shown in orange, FliG-bound MotAB shown blue. FliG and plug domains not modelled in 6YSF are transparent.

**Figure 4.**
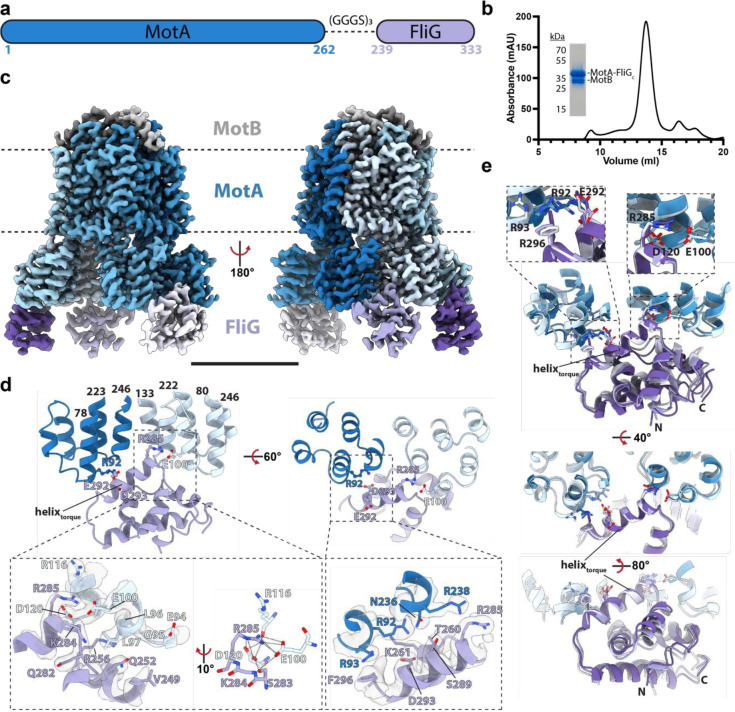
Design, purification, and cryo-EM structure of a stator complex bound to FliG_c_. **a,** Schematic of the *Clostridium sporogenes* MotA-FliG fusion construct used in this study. **b,** Size-exclusion chromatogram of MotAB-FliG_c_ complex with corresponding SDS-PAGE gel. **c,** Cryo-EM volume of MotAB-FliG_c_ complex. Volume for MotA, MotB and FliG_c_ subunits are depicted in various shades of blue, grey, and purple, respectively. The membrane, assigned from the position of the detergent micelle, is depicted by the dashed line. Scale bar is 50 Å. **d,** Modeling of a MotA-FliG interface. Cytoplasmic MotA extensions are shown in dark and light blue, and FliG_c_ is shown in purple. H-bonds are depicted as dashed lines. Cryo-EM density displayed as transparent surface. **e,** Structural alignment of the FliG_c_ torque helix across all bound FliG_c_ subunits. Subunit coloring consistent with panels b and c. Residues depicted in this figure are based on *C. sporogenes* annotation. See Table S1 for species equivalence in *E. coli* or *S*. typhimurium.

Both forms of the C-ring present the proposed “torque helix” of FliG_C_ at the top of the structure, directly underneath the inner membrane, primed for interaction with the stators (Fig 2c). To better understand the stator/C-ring interaction, we sought to solve the structure of the MotA_5_B_2_ complex with a FliG_C_ domain. An approach was taken in which the FliG_C_ domain was fused to the end of the MotA component via different lengths of unstructured linker sequence (Fig 4a,b). Several different linkers produced MotA structures with FliG_C_ domains docked between adjacent MotA monomers (Fig 4, Extended Data Fig. 11). Up to three FliG_C_ domains were observed bound to a single MotA_5_MotB_2_ complex, with the mode of docking broadly conserved in each case (Fig. 4d,e) and with no significant rearrangement of the MotA_5_B_2_ components compared to our earlier structure without FliG_C_ bound (Extended Data Fig. 12). All stator complexes are inherently asymmetric, with varying degrees of deviation from true C5 symmetry observed in the cytoplasmic domains of the MotA. The *Clostridium sporogenes* stator complex used here is the most asymmetric observed, and the FliG_C_ domains are only seen to bind between the closely spaced MotA pairs (Fig 4c), i.e. the pincer grip of a pair of adjacent MotA domains is required for the binding of the FliG_C_. The interaction sandwiches the FliG_C_ domain between the MotA cytoplasmic domains and presents the torque helix into the cavity (Fig. 4d,e). Conserved charged residues at either end of the torque helix, that have been previously implicated in torque generation, form interactions with conserved residues on adjacent MotAs, contributing to the pincer grip of the C-ring domain (Fig 4d,e).

## Model of rotational switching

**Figure 5.**
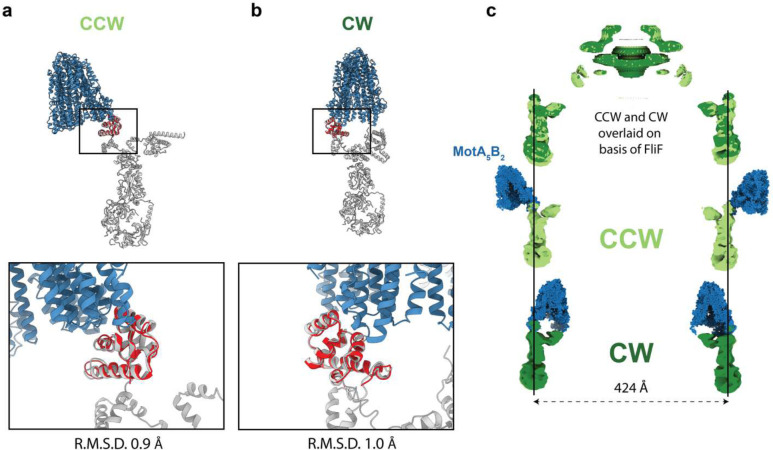
Overlaying MotA_5_B_2_-FliG on the CCW and CW C-rings reveals the stator complex switches from outside the C-ring to inside it. **a,b** The MotA_5_B_2_-FliG complex is overlaid on the same FliG_C_ domain with the CCW (a) and CW (b) C-ring subunits. The zoom box shows that the FliG_C_ domain is not structurally altered in the context of the C-ring subunit and overlays well, however, the rotation of the FliG_C_ domain in the context of the different rotational states results in the MotA_5_B2 complex being presented on the external (CCW) or internal (CW) face of the C-ring. **c,** Aligning the CCW and CW structures based on the invariant FliF component of the rotor reveals that (i) the Salmonella C-ring does not change diameter as part of switching and (upper panel shows the overlaid volumes with CCW – light green and CW – dark green (ii) The lower two panels show the MotA_5_B_2_-FliG_c_ complex in the context of the CCW and CW C-rings further illustrating that the stators move from pushing the C-ring on the outside to the inside of the C-ring. Thus explaining how a stator that rotates in a single direction can power bi-directional movement of the C-ring

The FliG_C_ domain is common to all three structures reported here. This therefore allowed us to construct models of the CCW and CW C-rings in complex with stators, via superposition of the shared domain (Fig. 5). The remarkable conformational changes observed between the CCW and CW states lead to a hugely different positioning of the stator. In the CCW state, the MotA_5_B_2_ complex sits outside the C-ring (Fig. 5a,c). This allows the clockwise rotation of the MotA subunits to rotate the FliG_C_ domains at the top of the C-ring in a counter-clockwise direction. The 180 degree rotation of the FliG_C_ domain on transitioning to the CW state means the stator would instead dock on the inside of the C-ring (Fig 5b,c). Here the clockwise rotation of the MotA subunits would rotate the C-ring in the clockwise direction. The overall dimensions of the two states are equivalent, even at the point of the FliG_C_, meaning that switching requires the stator to move closer to the basal body in the CW state (Fig. 5c). This is in contrast to previous models of switching, based on tomograms^21,22^, that suggested that the stators remain fixed in position and that the FliG_C_ domains move to a larger diameter during switching to the CW state. Whether this reflects a genuine difference between species with different size C-rings remains to be tested.

**Figure 6.**
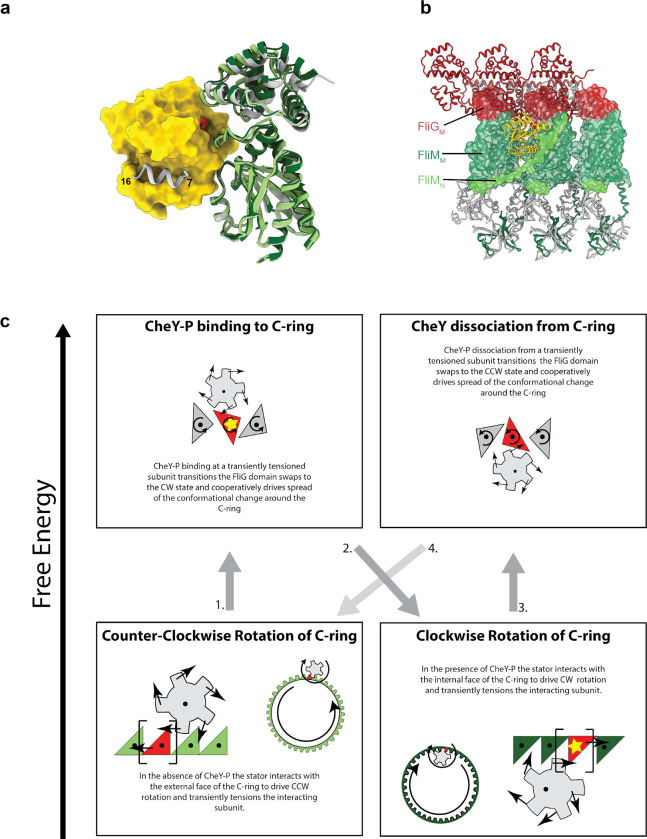
Mechanism for switching based on structures for CCW and CW flagellar C-rings **a,** Using AlphaFold2 to predict the interactions between CheY-P (yellow surface, phosphate shown as space filling atoms)and the C-ring (silver cartoon) generates an arrangement of the FliM_M_/FliG_M_ domains that matches that seen in the CW state (dark green) but not the CCW state (light green, overlaid based on FliM_M_ to highlight relative movement of FliG_M_). The previously determined interaction between the FliM N-terminus (silver helix on CheY-P surface) and CheY-P is highlighted. **b,** Mapping the location of CheY-P and the full N-terminus of FliM from the AlphaFold model to a set of three neighboring CW subunits reveals the nestling of CheY-P into the C-ring at a subunit-subunit interface and with interactions spread across two subunits. **c,** Schematic illustrating the mechanistic implications of our structures and models for how the uni-directional stators drive bi-directional rotation and its control by CheY-P binding.

Under physiological conditions, switching from the CCW to the CW state is triggered by binding of phosphorylated CheY to the FliM component of the C-ring^34^. CheY-P has been shown to bind to the extreme N-terminus of FliM^35^, in a portion of the structure that is disordered in both the CCW and CW states. However, this binding event has been proposed to act as a tether that allows binding at other sites on the C-ring^25^. In order to assess this possibility, we ran AlphaFold2.0 with various combinations of FliG, FliM and FliN domains. All combinations that involved the FliM_M_ domain produced models with a consistent CheY binding interface at the top of FliM_M_, with the phosphorylated residues (D57) pointing towards the FliG_M_:FliM_M_ interface (Fig. 6a,b). This binding mode is compatible with also binding the FliM N-terminus, as seen in crystal structures (Fig. 6a), and contains residues seen to undergo chemical shift in NMR binding studies in the interface^36^. Interestingly, overlay of the different structures via the FliM_M_ domain (Fig 6a) demonstrated that the FliG_M_ domain of the CCW state is shifted approximately 4 Å compared to the CW state. AlphaFold2 models of CheY bound to FliG_M_:FliM_M_ produced an interface consistent with the CW structure, suggesting a mechanism where CheY binding stabilizes the CW state (Fig 6a,b).

Combining our new structures and modelling of the CheY-P binding allows us to suggest the molecular basis for transfer of energy from the stators to the C-ring to achieve rotation and the mechanism by which directional switching occurs (Fig 6c). The key observation is that the laterally diffusible stators are reoriented in the two states such that their constant direction of rotation can drive the C-ring bidirectionally. The structural changes we observe between the states imply an active role for the stator complexes in the switching mechanism. Clockwise rotation of the MotAs against the CCW state of the C-ring pushes FliG_C_ in the direction it would need to travel to switch, i.e. under conditions of rotation there is a constant tension in the direction of switching. As the force applied to FliG is in the direction of the observed shift at the FliG_M_:FliM_M_ interface, CheY-P may lock the interface in this conformation once the stator acts. This fits with data that shows that CheY-P binding is favored under conditions of increased stator recruitment^37^. The rotation of the MotAs would also cause rotation of FliG_C_ away from the lateral locking against the neighboring subunits, again priming the system for switching in the right circumstance. The intricate domain swapping explains the cooperativity of the switching via conformational spread^38^; once one FliG starts to move it takes the neighboring copy with it, which would then propagate around the ring. Failure to perform this concerted motion would lead to clashes between neighboring subunits (Extended Data Fig. 6). Increased occupancy of CheY binding sites on FliM would then lock the C-ring in the CW structural state, triggering relocation of the stator complexes to the inner wall of the C-ring, and thereby driving clockwise rotation of the C-ring with the stator. As CheY-P levels in the cell drop, dissociation of CheY-P, and stator-driven pushing of the C-ring subunits in the direction of the CCW conformation, will lead to switching back to CCW structural state. Our structures therefore highlight both the complexity and relative simplicity of this remarkable molecular machine.

## Methods

### Bacterial strains and plasmids

The bacterial strains and plasmids used in this study are listed in Supplementary Table 1. Plasmids were generated by Gibson assembly of PCR fragments using the NEBuilder HiFi Master Mix (New England Biolabs). Fragments were created by PCR with the relevant primers (listed in Supplementary Table 2) using Q5 High-Fidelity DNA Polymerase (New England Biolabs) and genomic DNA templates obtained from *C. sporogenes* 388 (DSM 795). Gibson assembly and PCR were carried out according to the manufacturer’s recommendations.

### Basal body purification

The purification of basal bodies from *S.* Typhimurium strain TH25631 (CW locked) was described previously ^39^. Briefly, cells were incubated at 37°C, 200 rpm until an OD_600_ of 0.9–1, was reached, harvested by centrifugation, and resuspended in ice-cold sucrose solution for spheroplasting. Spheroplasts were lysed using 1% (v/v) Triton X-100 and basal bodies were collected by centrifugation. The pellet was resuspended in 2 mL of TET buffer (10 mM Tris pH 8, 5 mM EDTA pH 8, 0.1% (v/v) Triton X-100) then loaded onto 20–50% (w/w) sucrose gradients in 10 mM Tris pH 8, 5 mM EDTA pH 8, 0.03% (v/v) Triton X-100, made with a BioComp Gradient Station. Sucrose gradients were centrifuged for 14 h at 60,000×g, 4°C, fractionated, and fractions containing basal bodies were pooled and dialysed against 10 mM Tris pH 8, 5 mM EDTA pH 8, 0.03% (v/v) Triton X-100. Dialysed basal bodies were then concentrated to an A_280_ of 1.5, using a 300 kDa MWCO Nanosep^®^ centrifugal concentrator (PALL). The purification of basal bodies from *S.* Typhimurium strain TH25455 (CCW locked) was performed in an identical manner and basal bodies were concentrated to an A_280_ of 1.7.

### Purification of MotAB-FliG fusions

*C. sporogenes* MotA1–262-FliG239–333-MotB was expressed in *E. coli* MT56 from a pT12 vector encoding a C-terminal Twin-Strep tag on MotB. Cells were grown at 37 °C for 13 h in terrific broth medium containing kanamycin (50 μg ml−1) and L-rhamnose monohydrate (0.1% w/v) then collected by centrifugation at 4,000 g. Cell pellets were resuspended in Tris-buffered saline (TBS) (100 mM of Tris, 150 mM of NaCl, 1 mM of EDTA pH 8.0) plus 30 μg ml−1 of DNase I and 400 μg ml−1 of lysozyme for 30 min before passage through an EmulsiFlex-C3 homogenizer (Avestin) at 15,000 psi. Unbroken cells were removed by centrifugation at 24,000 g for 20 min. The supernatant was recovered and total membranes were collected by centrifugation at 200,000 g for 2 h. Membranes were resuspended in TBS and solubilized by incubation with 1% (w/v) lauryl maltose neopentyl glycol (LMNG; Anatrace) for 1 h. Insoluble material was removed by centrifugation at 100,000 g for 30 min. Solubilized membranes were then applied to a Strep-Tactin XT 4flow column (IBA). The resin was washed with 10 column volumes of TBS containing 0.02% (w/v) LMNG and proteins were eluted in 4 column volumes of TBS supplemented with 0.01% (w/v) LMNG and 50 mM of D-biotin (IBA). Eluates were concentrated using a 100-kDa molecular weight cut-off (MWCO) Vivaspin 6 (GE Healthcare) centrifugal filter unit and injected onto a Superose 6 Increase 10/300 GL size-exclusion column (GE Healthcare) pre-equilibrated in TBS plus 0.02% (w/v) LMNG. Peak fractions were collected and concentrated using a 100-kDa MWCO Vivaspin 500 (GE Healthcare) centrifugal filter unit.

### Cryo-EM sample preparation and imaging

Basal body cryo-EM grids were prepared using a Vitrobot Mark IV system (FEI) at a temperature of 4°C and 100% humidity. Basal body samples were applied to graphene oxide coated ^40^ Quantifoil Cu 300 mesh R 2/1 grids for 60 s before being blotted for 3 s, force −5 and then plunged into liquid ethane. Data were collected in counted super-resolution mode on a Titan Krios G3 (FEI) operating at 300 kV with a BioQuantum imaging filter (Gatan) and K3 direct detection camera (Gatan) using a physical pixel size of 0.832 Å. The imaging of basal bodies from *S.* Typhimurium strain TH25631 (CW locked) was described previously ^39^. Basal bodies from *S.* Typhimurium strain TH25455 (CCW locked) were collected with the same regime. 23,618 movies were collected at a dose rate of 14.5 e−/pix/s, exposure of 2.80 s, and total dose of 58.5 e−/Å2 over 40 fractions.

Four microliters of purified MotAB-FliGc at an A280nm of 1.3 was adsorbed onto glow-discharged holey carbon-coated grids (Quantifoil 300 mesh, Au R1.2/1.3) for 10 s. Grids were then blotted for 3 s at 100% humidity at 11 °C and frozen in liquid ethane using a Vitrobot Mark IV (Thermo Fisher Scientific). Data were collected in counted mode in EER format on a CFEG-equipped Titan Krios G4 (Thermo Fisher Scientific) operating at 300 kV with a Selectris X imaging filter (Thermo Fisher Scientific) with slit width of 10 e-V and Falcon 4 direct detection camera (Thermo Fisher Scientific) at ×165,000 magnification, with a physical pixel size of 0.693 Å. Movies were recorded at a dose rate of 14.2 e−/Å2/s and 3.98 s exposure for a total dose of 56.5 e−/Å2.

### Basal body cryo-EM data processing

Micrographs were processed in real time using the SIMPLE pipeline^41^, using SIMPLE-unblur for motion correction, SIMPLE-CTFFIND for CTF estimation and SIMPLE-picker for particle picking. After initial 2D classification in SIMPLE using cleanup2D to remove junk particles, all subsequent processing was performed in either RELION-3.1 3^42^ or cryoSPARC 4.2.1^43^ using the csparc2star.py script within UCSF pyem ^44^ to convert between formats. Global resolution estimates were derived from gold-standard Fourier shell correlations (FSCs) using the 0.143 criterion and local resolution estimation using an FSC threshold of 0.5, both within cryoSPARC. The workflow for cryo-EM image processing of the CCW-locked basal body C-ring is shown in Extended Data Fig. 1. Following 2D classification and selection of good classes in SIMPLE and Relion3.1, 61594 particles were re-extracted from the micrographs such that the C-ring was at the center of a 512 X 512 box (pixel size of 0.832 Å). Particles were subjected to *ab initio* reconstruction (k=1) in cryoSPARC with C34 symmetry applied to match the dominant species of the MS-ring. The resulting volume was low pass filtered to 20 Å and used as a reference for non-uniform refinement in cryoSPARC to generate volumes with C31-C37 symmetry. These volumes were used as references for heterogeneous refinement in C1 symmetry, and classes which lacked discernable basal body structure were discarded. Particles from the C33-C36 classes were then subjected to 2D classification in cryoSPARC, and only classes with countable top-down symmetries (C33-C35) were taken forward (Extended Data Fig. 3). A further round of heterogeneous refinement was carried out with C33-C35 references, and particles from the C34 class (15952 particles) were taken into non-uniform refinement in cryoSPARC with C34 symmetry applied, producing a 4.6 Å map which allowed docking of AlphaFold2 ^45^ models of the protein domains. These were used to create a model of three adjacent C-ring “subunits” for purposes of creating a mask. The particles were then symmetry expanded with the C34 symmetry, and Local Refinement was carried out in cryoSPARC in C1 symmetry within the trimer mask. This produced a map with a global resolution of 3.6 Å, and local resolution estimates as high as 3.2 Å, with clear sidechain density in the FliM and FliN components. The workflow for cryo-EM image processing of the CW-locked basal body C-ring is shown in Extended Data Fig. 2. Following 2D classification and selection of good classes in SIMPLE and Relion3.1, 117152 particles were re-extracted from the micrographs such that the C-ring was at the center of a 512 X 512 box (pixel size of 0.832 Å). Particles were subjected to *ab initio* reconstruction (k=1) in cryoSPARC with C34 symmetry applied to match the dominant species of the MS-ring. The resulting volume was low pass filtered to 20 Å and used as a reference for non-uniform refinement in cryoSPARC to generate volumes with C31-C37 symmetry. These volumes were used as references for heterogeneous refinement in C1 symmetry, and classes which lacked discernable basal body structure were discarded. Particles from the C33-C36 classes were then subjected to 2D classification in cryoSPARC, and only classes with countable top-down symmetries (C33-C35) were taken forward (Extended Data Fig. 3). A further round of heterogeneous refinement was carried out with C33-C35 references, and particles from the C34 class (19363 particles) were taken into non-uniform refinement in cryoSPARC with C34 symmetry applied, producing a 5.4 Å map which allowed docking of AlphaFold2 models of the protein domains. These were used to create a model of three adjacent C-ring “subunits” for purposes of creating a mask. The particles were then symmetry expanded with the C34 symmetry, and Local Refinement was carried out in cryoSPARC in C1 symmetry within the trimer mask. This produced a map with a global resolution of 4.0 Å, and local resolution estimates as high as 3.3 Å, with clear sidechain density in the FliM and FliN components.

### MotAB-FliG fusion cryo-EM data processing

Movie preprocessing was performed in real time using the SIMPLE 3.0 pipeline 1, using SIMPLE-unblur for patched (20 × 20) motion correction, SIMPLE-CTFFIND for patched CTF estimation and SIMPLE-picker for particle picking. After initial 2D classification in SIMPLE using cleanup2D to remove junk particles, all subsequent processing was performed in either cryoSPARC 2 or RELION-3.1 3 using the csparc2star.py script within UCSF pyem to convert between formats 4. Global resolution estimates were derived from gold-standard Fourier shell correlations (FSCs) using the 0.143 criterion and local resolution estimation using an FSC threshold of 0.5, both within cryoSPARC. The workflow for cryo-EM image processing of the MotAB-FliGc complex is shown in Extended Data Fig. 11. Briefly, 49,077 movies were collected and 5,264,890 particles extracted from motion-corrected micrographs then subjected to reference-free 2D classification in SIMPLE (k=300) followed by an additional round of 2D classification in cryoSPARC (k=200) both using a 180 Å diameter soft spherical mask. Selected particles (1,551,143) were subjected to multi-class *ab initio* reconstructions (k=5) in cryoSPARC. Representative volumes (junk, monomeric stator complex, dimeric stator complex assembly) were subsequently lowpass-filtered to 20 Å and used as references for heterogeneous refinement against the full particle selection (1,551,143 particles) in cryoSPARC. Non-uniform refinement in cryoSPARC was then performed using particles belonging to dimeric stator complex assembly class (corresponding to 77.8 % of total particles) against a corresponding volume lowpass-filtered to 30 Å, generating a 2.8 Å map. Particles were then re-extracted with re-centering based on the center of mass of each stator complex within the dimeric assembly and then separately Bayesian polished within RELION. Polished particles were imported and volumes reconstructed in cryoSPARC using prior poses. Particle subtraction was performed for each particle set using a mask encompassing the off-center stator complex. Following this, both sets were combined and subjected to 2D classification (k=300) using a soft spherical mask of 160 Å in diameter to further remove low quality particles. Selected particles (1,761,086) were subjected to non-uniform refinement against one of the particle-subtracted volumes lowpass-filtered to 30 Å resulting in a 2.3 Å map with stronger density for FliGC. Because each individual stator complex within the dimeric assembly accommodates either 2 or 3 FliGc, alignment-free 3D classification (k=4) was performed in RELION using a mask covering the third FliGc binding site to separate bound and unbound forms. Particles belonging to the bound form classes (982,095 particles; 55.8% of total) were selected and non-uniform refined in cryoSPARC against the previous map lowpass-filtered to 30 Å, resulting in a 2.3 Å volume. Further alignment-free 3D classification (k=4) in RELION using a mask surrounding the transmembrane helices (TMHs) of MotB was performed to further improve map quality. Particles belonging to classes containing strong TMHs were combined (719,539 particles) and non-uniform refined against the preceding map lowpass-filtered to 15 Å yielding a 2.4 Å map. This map yielded the most complete and highest resolution density for the MotA-FliG interface. To improve the interpretability of side chains in regions of weaker density, deepEMhancer^46^ was used on the unfiltered half maps using the “highres” trained model.

### Model building and refinement

Atomic models were built using Coot v0.97^47^. AlphaFold2 ^45^ models of the domains of FliG, FliM and FliN were docked into the highest resolution maps, followed by iterative manual building and real-space refinement into unsharpened, sharpened, or deepEMhanced^46^ maps. The atomic model of MotAB-FliGc was generated first by rigid-body fitting our previously deposited *C. sporogenes* MotAB model (PDB: 6YSF) and the C-terminal domain of a *C. sporogenes* FliG model generated using PHYRE^48^ followed by iterative manual building and real-space refinement into unsharpened, sharpened (B-factor of −69), or deepEMhanced^46^ map within Coot v0.97^47^. Final real-space refinement into the B-factor sharpened map with rotamer and Ramachandran restraints was performed in PHENIX^49^. Models were validated using Molprobity^50^ within PHENIX. Cryo-EM data collection, image processing and structure refinement statistics are listed in Table 1. Interaction interfaces were analyzed using PDBePISA^51^. Figures were prepared using UCSF Chimera v1.1.5 or UCSF ChimeraX v.1.6.111. Figures displaying MotAB + FliGc volumes were generated with deepEMhancer maps to improve visualization of side chain density. Residue numbering of the MotAB-FliGc adopts the *C. sporogenes* sequence and model; a residue conversion table is provided (Supplementary Table 1).

## Extended Data

## Figures and Tables

**Table 1.** Cryo-EM data collection, refinement and validation statistics

	MotAB + FliG_c_	CCW	CW

**Data collection and processing**			
Magnification	165,000	165,000	165,000
Voltage (kV)	300	300	300
Electron exposure (e−/Å^2^)	56.5	58.5	58.5
Defocus range (pm)	−2.5 to −1.0	−2.0 to −0.5	−2.0 to −0.5
Pixel size (Å)	0.693	0.832	0.832
Symmetry imposed	C1	C1	C1
Initial particle images (no.)	5,264,890	61,594	117,152
Final particle images (no.)	719,539	15,952	19,363
Map resolution (Å)	2.4	3.6	4.0
FSC threshold	0.143	0.143	0.143
Map resolution range (Å)	2.0 – 36.5	3.2–4.5	3.3–5.2
**Refinement**			
Initial model used (PDB code)	6YSF		
Model resolution (Å)	2.4	3.6	4.0
FSC threshold	0.5	0.143	0.143
Map sharpening *B* factor (Å^2^)	−69.0	−99.0	−128.0
Model composition			
Non-hydrogen atoms	12605	7324	7193
Protein residues	1632	926	910
Ligands	N/A	N/A	N/A
*B* factors (Å^2^)			
Protein	40.7	150.1	185.2
Ligand	N/A	N/A	N/A
R.m.s. deviations			
Bond lengths (Å)	0.003	0.004	0.004
Bond angles (°)	0.485	1.245	0.912
Validation			
MolProbity score	1.65	2.67	2.81
Clashscore	8.78	19.3	23.0
Poor rotamers (%)	0.36	3.1	4.3
Ramachandran plot			
Favored (%)	96.96	91.1	92.1
Allowed (%)	3.04	8.1	7.5
Disallowed (%)	0.00	0.8	0.4
CC (mask)	0.81	0.71	0.68

## Data Availability

Cryo-EM volumes and atomic models have been deposited to the EMDB (accession codes EMDB-42139, 8UCS).
